# Long-term neurological symptoms after acute COVID-19 illness requiring hospitalization in adult patients: insights from the ISARIC-COVID-19 follow-up study

**DOI:** 10.1007/s00415-023-12133-y

**Published:** 2023-12-06

**Authors:** Denise Battaglini, Nicole M. White, Lavienraj Premraj, Barbara Wanjiru Citarella, Laura Merson, Chiara Robba, David Thomson, Sung-Min Cho, ISARIC Clinical Characterization Group, Laurent Abel, Amal Abrous, Kamal Abu Jabal, Manuella Ademnou, Younes Ait Tamlihat, Aliya Mohammed Alameen, Marta Alessi, Beatrice Alex, Kévin Alexandre, Adam Ali, Kazali Enagnon Alidjnou, Clotilde Allavena, Nathalie Allou, Pasquale Anania, Claire Andrejak, Andrea Angheben, François Angoulvant, Séverine Ansart, Jean-Benoît Arlet, Elise Artaud-Macari, Grace Assi, Jean Baptiste Assie, Fouda Atangana, Johann Auchabie, Hugues Aumaitre, Adrien Auvet, Eyvind W Axelsen, Laurène Azemar, Cecile Azoulay, Benjamin Bach, Delphine Bachelet, Claudine Badr, Roar Bævre-Jensen, John Kenneth Baillie, Firouzé Bani-Sadr, Wendy S Barclay, Marie Bartoli, Joaquín Baruch, Romain Basmaci, Jules Bauer, Alexandra Bedossa, Husna Begum, Sylvie Behilill, Anna Beltrame, Marine Beluze, Nicolas Benech, Delphine Bergeaud, José Luis Bernal Sobrino, Giulia Bertoli, Simon Bessis, Sybille Bevilcaqua, Karine Bezulier, Krishna Bhavsar, Sandra Bichoka, Zeno Bisoffi, Laurent Bitker, Mathieu Blot, Lucille Blumberg, Laetitia Bodenes, Debby Bogaert, Anne-Hélène Boivin, Ariel Bolanga, Isabela Bolaños, Pierre-Adrien Bolze, Raphaël Borie, Elisabeth Botelho-Nevers, Lila Bouadma, Olivier Bouchaud, Sabelline Bouchez, Damien Bouhour, Kévin Bouiller, Laurence Bouillet, Camile Bouisse, Anne-Sophie Boureau, Maude Bouscambert, Aurore Bousquet, Marielle Boyer-Besseyre, Axelle Braconnier, Sonja Hjellegjerde Brunvoll, Marielle Buisson, Danilo Buonsenso, Donald Buri, Aidan Burrell, Ingrid G Bustos, André Cabie, Eder Caceres, Cyril Cadoz, Jose Andres Calvache, Valentine Campana, Pauline Caraux-Paz, Nicolas Carlier, Thierry Carmoi, Marie-Christine Carret, Gail Carson, Maire-Laure Casanova, Guylaine Castor-Alexandre, François-Xavier Catherine, Minerva Cervantes-Gonzalez, Anissa Chair, Catherine Chakveatze, Meera Chand, Jean-Marc Chapplain, Charlotte Charpentier, Julie Chas, Léo Chenard, Antoine Cheret, Thibault Chiarabini, Catherine Chirouze, Bernard Cholley, Marie-Charlotte Chopin, Yock Ping Chow, Sara Clohisey, Gwenhaël Colin, Marie Connor, Anne Conrad, Graham S Cooke, Hugues Cordel, Andrea Cortegiani, Grégory Corvaisier, Camille Couffignal, Sandrine Couffin-Cadiergues, Roxane Courtois, Stéphanie Cousse, Juan Luis Cruz Bermúdez, Jaime Cruz Rojo, Elodie Curlier, Ana da SilvaFilipe, Charlene Da Silveira, Andrew Dagens, John Arne Dahl, Jo Dalton, Cammandji Damien, Etienne De Montmollin, Cristina De Rose, Thushan de Silva, Alexa Debard, Marie-Pierre Debray, Nathalie DeCastro, Romain Decours, Eve Defous, Isabelle Delacroix, Eric Delaveuve, Karen Delavigne, Christelle Delmas, Pierre Delobel, Elisa Demonchy, Emmanuelle Denis, Dominique Deplanque, Diane Descamps, Mathilde Desvallées, Alpha Diallo, Sylvain Diamantis, Fernanda Dias Da Silva, Kévin Didier, Jean-Luc Diehl, Jérôme Dimet, Vincent Dinot, Fara Diop, Alphonsine Diouf, Cedric Djadda, Félix Djossou, Annemarie B Docherty, Christl A Donnelly, Céline Dorival, Eric D’Ortenzio, Yash Doshi, Nathalie Dournon, Thomas Drake, Amiel A Dror, Murray Dryden, Vincent Dubee, François Dubos, Alexandre Ducancelle, Susanne Dudman, Paul Dunand, Jake Dunning, Bertrand Dussol, Xavier Duval, Anne Margarita Dyrhol-Riise, Ada Ebo, Michael Edelstein, Linn Margrete Eggesbø, Mohammed ElSanharawi, Brigitte Elharrar, Merete Ellingjord-Dale, Lauren Eloundou, Philippine Eloy, Isabelle Enderle, Gervais Eneli, Ilka Engelmann, Vincent Enouf, Olivier Epaulard, Hélène Esperou, Marina Esposito-Farese, Rachel Essaka, Manuel Etienne, Mirjam Evers, Marc Fabre, Isabelle Fabre, Cameron J Fairfield, Karine Faure, Raphaël Favory, François-Xavier Ferrand, Eglantine Ferrand Devouge, Nicolas Ferriere, Céline Ficko, William Finlayson, Thomas Flament, Tom Fletcher, Aline-Marie Florence, Berline Fotso, Erwan Fourn, Robert A Fowler, Christophe Fraser, Stéphanie Fry, Valérie Gaborieau, Rostane Gaci, Jean-Charles Gagnard, Amandine Gagneux-Brunon, Sérgio Gaião, Linda Gail Skeie, Carrol Gamble, Noelia García Barrio, Esteban Garcia-Gallo, Denis Garot, Valérie Garrait, Anatoliy Gavrylov, Alexandre Gaymard, Eva Geraud, Louis Gerbaud Morlaes, Jade Ghosn, Tristan Gigante, Guillermo Giordano, Michelle Girvan, Valérie Gissot, Daniel Glikman, François Goehringer, Kyle Gomez, Marie Gominet, Yanay Gorelik, Isabelle Gorenne, Laure Goubert, Cécile Goujard, Tiphaine Goulenok, Pascal Granier, Christopher A Green, William Greenhalf, Segolène Greffe, Fiona Griffiths, Jérémie Guedj, Martin Guego, Romain Guery, Anne Guillaumot, Laurent Guilleminault, Thomas Guimard, Ali Hachemi, Nadir Hadri, Matthew Hall, Sophie Halpin, Rebecca Hamidfar, Bato Hammarström, Hayley Hardwick, Ewen M Harrison, Janet Harrison, Lars Heggelund, Ross Hendry, Maxime Hentzien, Diana Hernandez, Liv Hesstvedt, Rupert Higgins, Haider Hirkani, Hikombo Hitoto, Antonia Ho, Alexandre Hoctin, Isabelle Hoffmann, Peter Horby, Ikram Houas, Jean-Sébastien Hulot, Samreen Ijaz, Patrick Imbert, Mariachiara Ippolito, Margaux Isnard, Danielle Jaafar, Salma Jaafoura, Julien Jabot, Clare Jackson, Waasila Jassat, Stéphane Jaureguiberry, Florence Jego, Synne Jenum, Cédric Joseph, Mercé Jourdain, Ouifiya Kafif, Florentia Kaguelidou, Sabina Kali, Deepjyoti Kalita, Christiana Kartsonaki, Seán Keating, Pulak Kedia, Sadie Kelly, Kalynn Kennon, Younes Kerroumi, Antoine Khalil, Krish Kherajani, Saye Khoo, Antoine Kimmoun, Eyrun Floerecke Kjetland Kjetland, Paul Klenerman, Stephen R Knight, Stephanie Kouba, Arsène Kpangon, Oksana Kruglova, Galyna Kutsyna, Sylvie Kwedi, Marie Lachatre, Marie Lacoste, Nadhem Lafhej, Marie Lagrange, Fabrice Laine, Olivier Lairez, Antonio Lalueza, Marc Lambert, Marie Langelot-Richard, Vincent Langlois, Cédric Laouénan, Samira Laribi, Delphine Lariviere, Stéphane Lasry, Sakshi Lath, Odile Launay, Didier Laureillard, Yoan Lavie-Badie, Andy Law, Minh Le, Clément Bihan, Cyril Bris, Georges Falher, Lucie Fevre, Quentin Hingrat, Marion Maréchal, Soizic Mestre, Gwenaël Moal, Vincent Moing, Hervé Nagard, James Lee, Jennifer Lee, Gary Leeming, Bénédicte Lefebvre, Laurent Lefebvre, Benjamin Lefèvre, Sylvie LeGac, Jean-Daniel Lelievre, Adrien Lemaignen, Véronique Lemee, Anthony Lemeur, Marc Leone, Quentin Lepiller, François-Xavier Lescure, Olivier Lesens, Mathieu Lesouhaitier, Sophie Letrou, Bruno Levy, Yves Levy, Claire Levy-Marchal, Erwan L’Her, Geoffrey Liegeon, Bruno Lina, Andreas Lind, Guillaume Lingas, Sylvie Lion-Daolio, Marine Livrozet, Paul Loubet, Bouchra Loufti, Guillame Louis, Miles Lunn, Liem Luong, Dominique Luton, Moïse Machado, Gabriel Macheda, Rafael Mahieu, Sophie Mahy, Mylène Maillet, Thomas Maitre, Denis Malvy, Victoria Manda, Laurent Mandelbrot, Julie Mankikian, Aldric Manuel, Samuel Markowicz, Laura Marsh, John Marshall, Guillaume Martin-Blondel, Martin Martinot, Olga Martynenko, Moise Massoma, Palmer Masumbe, Mathieu Mattei, Laurence Maulin, Thierry Mazzoni, Colin McArthur, Sarah E McDonald, Kenneth A McLean, Cécile Mear-Passard, Nastia Medombou, France Mentré, Alexander J Mentzer, Emmanuelle Mercier, Noémie Mercier, Antoine Merckx, Mayka Mergeay-Fabre, Roberta Meta, Agnès Meybeck, Alison M Meynert, Vanina Meysonnier, Mehdi Mezidi, Céline Michelanglei, Isabelle Michelet, Shona C Moore, Sarah Moore, Lucia Moro, Hugo Mouquet, Julien Moyet, Caroline Mudara, Jimmy Mullaert, Fredrik Müller, Marlène Murris, Srinivas Murthy, Alamin Mustafa, Nadège Neant, Anthony Nghi, Duc Nguyen, Nerissa Niba, Alistair D Nichol, Mahdad Noursadeghi, Saad Nseir, Leonard Numfor, Elsa Nyamankolly, Piero L Olliaro, Pierre Ondobo, Wilna Oosthuyzen, Peter Openshaw, Paul Otiku, Nadia Ouamara, Rachida Ouissa, Eric Oziol, Maïder Pagadoy, Justine Pages, Massimo Palmarini, Nathalie Pansu, Aurélie Papadopoulos, Rachael Parke, Jérémie Pasquier, Bruno Pastene, Drashti Patel, Christelle Paul, William A Paxton, Jean-François Payen, Florent Peelman, Nathan Peiffer-Smadja, Vincent Peigne, Daniel Perez, Thomas Perpoint, Vincent Pestre, Ventzislava Petrov-Sanchez, Gilles Peytavin, Walter Picard, Olivier Picone, Lionel Piroth, Chiara Piubelli, Riinu Pius, Laurent Plantier, Julien Poissy, Ryadh Pokeerbux, Georgios Pollakis, Diane Ponscarme, Sébastien Preau, Mark G Pritchard, Else Quist-Paulsen, Christian Rabaud, Marie Rafiq, Blandine Rammaert, Christophe Rapp, Stanislas Rebaudet, Sarah Redl, Martine Remy, Anne-Sophie Resseguier, Matthieu Revest, Antonia Ricchiuto, Laurent Richier, Patrick Rispal, Karine Risso, Stephanie Roberts, David L Robertson, Olivier Robineau, Paola Rodari, Pierre-Marie Roger, Amanda Rojek, Mélanie Roriz, Manuel Rosa-Calatrava, Andrea Rossanese, Patrick Rossignol, Carine Roy, Benoît Roze, Clark D Russell, Nadia Saidani, Hélène Salvator, Olivier Sanchez, Vanessa Sancho-Shimizu, Pierre-François Sandrine, Oana Săndulescu, Benjamine Sarton, Ankana Satya, Egle Saviciute, Arnaud Scherpereel, Marion Schneider, Janet T Scott, James Scott-Brown, Nicholas Sedillot, Malcolm G Semple, Eric Senneville, Catherine A Shaw, Victoria Shaw, Rohan Shetty, Jeanne Sibiude, Louise Sigfrid, Dario Sinatti, Girish Sindhwani, Mahendra Singh, Vegard Skogen, Sue Smith, Tom Solomon, Agnès Sommet, Arne Søraas, Albert Sotto, Edouard Soum, Elisabetta Spinuzza, Shiranee Sriskandan, Sarah Stabler, Trude Steinsvik, Birgitte Stiksrud, Adrian Streinu-Cercel, Anca Streinu-Cercel, David Stuart, Richa Su, Charlotte Summers, Lysa Tagherset, Renaud Tamisier, Coralie Tardivon, Pierre Tattevin, Marie-Capucine Tellier, François Téoulé, Olivier Terrier, Nicolas Terzi, Vincent Thibault, Simon-Djamel Thiberville, Benoît Thill, Emma C Thomson, Mathew Thorpe, Ryan S Thwaites, Vadim Tieroshyn, Jean-François Timsit, Noémie Tissot, Kristian Tonby, Cécile Tromeur, Tiffany Trouillon, Jeanne Truong, Christelle Tual, Sarah Tubiana, Jean-Marie Turmel, Lance C. W. Turtle, Anders Tveita, Timothy M Uyeki, Piero Valentini, Sylvie Van Der Werf, Noémie Vanel, Charline Vauchy, Aurélie Veislinger, Gayatri Vishwanathan, Benoit Visseaux, Fanny Vuotto, Steve Webb, Jia Wei, Murray Wham, Paul Henri Wicky, Aurélie Wiedemann, Natalie Wright, Yazdan Yazdanpanah, Cécile Yelnik, Hodane Yonis, Marion Zabbe, Maria Zambon, Hiba Zayyad, David Zucma.

**Affiliations:** 1 IRCCS Ospedale Policlinico San Martino, Genova, Italy; 2 Australian Centre for Health Services Innovation and Centre for Healthcare Transformation, School of Public Health & Social Work, Queensland University of Technology, Brisbane, Australia; 3 Griffith University School of Medicine, Gold Coast, Brisbane, Australia; 4 Critical Care Research Group, The Prince Charles Hospital, Brisbane, Australia; 5 International Severe Acute Respiratory and emerging Infections Consortium (ISARIC), Pandemic Sciences Institute, University of Oxford, Oxford, UK; 6 Department of Surgical sciences and Integrated Diagnostics, University of Genoa, Genova, Italy; 7 Department of Anesthesia and Perioperative Medicine, University of Cape Town, Cape Town, South Africa; 8 Division of Critical Care, Groote Schuur Hospital, Cape Town, South Africa; 9 Neuroscience Critical Care Division, Departments of Neurology, Surgery, and Anaesthesiology and Critical Care Medicine, Johns Hopkins University School of Medicine, Baltimore, Maryland USA

Dear Editor,

Coronavirus disease-2019 (COVID-19) has devastated healthcare systems and public health globally [1]. Many patients develop a wide spectrum of persisting or new symptoms 3 months after the acute COVID-19 illness (long-COVID-19), and these symptoms can persist for at least 2 months [2, 3]. There is significant variability in the definitions with the lack of standardization and hence the reported frequency of long-COVID-19 also varies.

Furthermore, there is sparser data with a significant heterogeneity on neurological long-COVID-19 symptoms [4].

Neurological manifestations represent a possible presentation of long-COVID-19 [5–7]. Data on the type of symptoms and prevalence of neurological long-COVID-19 are still in evolution [1, 5]. Hence, a clear understanding of neurological long-COVID-19 would aid healthcare systems in implementing health resources to measure and manage this global healthcare burden.

Herein, in this study we aimed to characterize the type and prevalence of neurological symptoms related to neurological long-COVID-19 from a large international multicenter cohort of adults after discharge from hospital for acute COVID-19.

This is an international, multicenter, prospective, observational cohort using the ISARIC WHO COVID-19 Clinical Characterization Protocol, approved by the WHO Ethics Committee (RPC571 and RPC572).

Local Ethics approval was obtained from participating centers according to local regulatory rules as appropriate.

Inclusion criteria were*:* patients ≥ 18 years-old; patients previously admitted to hospital with COVID-19; follow-up data available at least 1-month post- discharge from hospital or health center; person (or family member/next of kin for patients who lack capacity) consent to participate.

The case report form (CRF) was completed as a patient self-assessment through an online link, telephone interview, or in-clinic. Data were collected as first presentation of symptoms and persistent presentation. First presentation described participants who did not have neurological symptoms at hospitalization. Persistent presentation is persistence of symptoms among those who had neurological symptoms evaluated at initial hospitalization. Survey follow-up was defined as three monthly intervals from post-discharge follow-up, up to 12 months. Six main neurological symptoms were collected during hospitalization and follow-up. Additional neurological symptoms were collected at follow-up only. Specifics of survey schematic overview of the follow-up data time frame are reported in *Supplementary Material (SM) Item S1*. Follow-up asking for new or persistent neurological symptoms has been performed by phone call or in-person interview at each time point: 1–3 months, 4–6 months, 7–9 months, and 10–12 months.

Main neurological symptoms included confusion, anosmia, ageusia, fatigue/malaise, muscle aches/joint pain, and seizures. Additional neurological symptoms included dizziness, erectile dysfunction, fainting/ blackouts, headache, loss of sensation, muscle weakness, paresthesia, problems seeing, problems speaking or communicating, problems swallowing or chewing, problems with balance, tinnitus, and tremors.

Observed prevalence of neurological symptoms were estimated based on survey follow-up time, age at disease onset and sex. Unadjusted symptom prevalence by survey follow-up period was summarized as percentages with 95% confidence intervals (CIs), assuming a Gamma distribution. Symptom prevalence estimates were stratified by initial versus repeat follow-up assessment. Period prevalence by follow-up was also calculated. Symptom prevalence by age and sex was examined by logistic regression. Regression models with neurological symptoms as an outcome included fixed effects for sex and age, and their interaction, nested within the survey follow-up period. Age was modelled by polynomial terms up to order of 3. Analyses were completed in R using the lmerTest package.

Overall, 11,357 adults (median age = 56 (IQR = 45 to 67) years; 42% female) with acute COVID-19 hospitalization from January 2020 to December 2022 were analyzed (*SM Item S2*). Frequencies are stratified by the availability of neurological signs and symptoms evaluated at disease onset/hospital admission as shown in *SM Item S3*. Baseline characteristics are presented in *SM Item S4*.

Fatigue/malaise was the most frequent neurological manifestation reported at acute hospitalization with 54.9% (95%CI 53.6–56.2%), followed by muscle aches/joint pain 35.8% (95%CI 34.5–37.0%), ageusia 20.7% (95%CI 19.6–21.8%), anosmia 18.3% (95%CI 17.2–19.3%), confusion 7.9% (95%CI 7.1–8.7%), and seizures 0.9% (95%CI 0.5–1.2%).

More than half (55.3%) of participants had one or more neurological symptoms during their hospitalization. At follow-up, first presentation of symptoms was found in 40.6% (95%CI = 38.0–43.4%) at 1–3 months, 39.1% (95%CI = 36.5–41.9%) at 4–6 months, 23.1% (95%CI = 19.0–27.8%) at 7–9 months, and 4.4% (95%CI = 2.4–7.4%) at 10–12 months had 1 or more neurological symptoms. Persistent presentation of symptoms was found in 53.3% (95%CI = 50.6–56.1%) at 1–3 months, 58.4% (95%CI = 56.0–60.9%) at 4–6 months, 55.4% (95%CI = 52.1–58.8%) at 7–9 months, and 40.4% (95%CI = 37.3–42.8%) at 10–12 months had 1 or more neurological symptoms.

At 1–3 months, estimates of first presentation of symptoms were: fatigue/malaise 51.8% (95%CI = 48.0–55.9%), muscle aches/joint pain 18.6% (95%CI = 16.9–20.4%), ageusia 5.2% (95%CI = 4.1–6.4%), anosmia 4.1% (95%CI = 3.1–5.2%), and confusion 10.9% (95%CI = 9.6–12.4%). Estimates of persistent presentation of symptoms were: fatigue/malaise 41.9% (95%CI = 39.4–44.5%), muscle aches/joint pain 27.6% (95%CI = 25.6–29.7%), ageusia 7.9% (95%CI = 6.8–9.2%), anosmia 7.7% (95%CI = 6.6–8.9%), and confusion 19.4% (95%CI = 17.2–21.7%).

At 4–6 months, estimates for first presentation of symptoms were: fatigue/malaise 50.8% (95%CI = 46.9–54.9%), muscle aches/joint pain 19.4% (95%CI = 17.6–21.3%), ageusia 3.5% (95%CI = 2.6–4.7%), anosmia 4.4% (95%CI = 3.3–5.6%), and confusion 11.5% (95%CI = 10.1–13.1%). Estimates of persistent presentation of symptoms were: fatigue/malaise 44.3% (95%CI = 42.2–46.5%), muscle aches/ joint pain 34.2% (95%CI = 32.3–36.1%), ageusia 6.7% (95%CI = 5.9–7.6%), anosmia 6.9% (95%CI = 6.1–7.8%), and confusion 29.4% (95%CI = 27.3–31.6%).

At 7–9 months, estimates of first presentation of symptoms were: fatigue/malaise 26.1% (95%CI = 20.5–32.8%), muscle aches/joint pain 20.5% (95%CI = 16.9–24.7%), ageusia 3.9% (95%CI = 2.1–6.6%), anosmia 4.1% (95%CI = 2.3–6.9%), and confusion 9.4% (95%CI = 7.0–12.5%). Estimates of persistent presentation of symptoms were: fatigue/malaise 43.6% (95%CI = 40.6–46.7%), muscle aches/joint pain 30.1% (95%CI = 27.6–32.7%), ageusia 6.2% (95%CI = 5.1–7.5%), anosmia 7.1% (95%CI = 5.9–8.4%), and confusion 26.6% (95%CI = 23.9–29.5%).

At 10–12 months, estimates of the first presentation of symptoms were: fatigue/malaise 3.1% (95%CI = 1.4–5.8%), muscle aches/joint pain 2.5% (95%CI = 1.1–4.9%), ageusia and anosmia 0.0%, and confusion in 2.2% (95%CI = 0.9–4.5%). Estimates of persistent presentation of symptoms were: fatigue/malaise 29.9% (95%CI = 27.6–32.4%), muscle aches/joint pain 23.1% (95%CI = 21.0–25.2%), ageusia 3.3% (95%CI = 2.6–4.2%), anosmia 3.9% (95%CI = 3.1–4.9%), and confusion 16.9% (95%CI = 15.2–18.8%).

Table 1 shows estimates of first and persistent presentation of symptoms. *SM Item S5* shows period prevalence of neurological symptoms post-hospital discharge. Additional symptoms assessed by survey only, not evaluated at acute hospitalization, are shown in *SM Item S6*. Missing data points were excluded from the analysis of each symptom. Trajectories of prevalence of neurological symptoms post-hospital discharge between males and females are shown in Fig. 1. *SM Item S7* shows trajectories of prevalence of neurological symptoms post-acute onset of COVID-19 between males and females.

**Table 1:** Observed prevalence and persistence of neurological symptoms over survey follow-up post hospital discharge

Symptom*	Hospitalization	1 – 3 months		4 – 6 months		7 – 9 months		10 – 12 months	
	First presentation of symptom	First presentation of symptom	Repeat presentation of symptom	First presentation of symptom	Repeat presentation of symptom	First presentation of symptom	Repeat presentation of symptom	First presentation of symptom	Repeat presentation of symptom
Confusion	7.9% (7.1% to 8.7%) 331/4190	10.9% (9.6% to 12.4%) 242/2217	19.4% (17.2% to 21.7%) 289/1493	11.5% (10.1% to 13.1%) 242/2103	29.4% (27.3% to 31.6%) 761/2589	9.4% (7.0% to 12.5%) 49/520	26.6% (23.9% to 29.5%) 351/1319	2.2% (0.9% to 4.5%) 7/323	16.9% (15.2% to 18.8%) 342/2022
Fatigue/malaise	54.9% (53.6% to 56.2%) 3172/5781	51.8% (48.0% to 55.9%) 687/1325	41.9% (39.4% to 44.5%) 1040/2483	50.8% (46.9% to 54.9%) 629/1239	44.3% (42.2% to 46.5%) 1641/3702	26.1% (20.5% to 32.8%) 74/283	43.6% (40.6% to 46.7%) 794/1822	3.1% (1.4% to 5.8%) 9/295	29.9% (27.6% to 32.4%) 618/2067
Anosmia	18.3% (17.2% to 19.3%) 964/5280	4.1% (3.1% to 5.2%) 64/1574	7.7% (6.6% to 8.9%) 173/2252	4.4% (3.3% to 5.6%) 61/1397	6.9% (6.1% to 7.8%) 245/3544	4.1% (2.3% to 6.9%) 14/339	7.1% (5.9% to 8.4%) 124/1756	0.0% (–) 0/305	3.9% (3.1% to 4.9%) 81/2058
Ageusia	20.7% (19.6% to 21.8%) 1092/5271	5.2% (4.1% to 6.4%) 81/1568	7.9% (6.8% to 9.2%) 178/2252	3.5% (2.6% to 4.7%) 50/1409	6.7% (5.9% to 7.6%) 237/3531	3.9% (2.1% to 6.6%) 13/336	6.2% (5.1% to 7.5%) 109/1756	0.0% (–) 0/305	3.3% (2.6% to 4.2%) 68/2053
Muscle aches/joint pain	35.8% (34.5% to 37.0%) 2060/5761	18.6% (16.9% to 20.4%) 439/2366	27.6% (25.6% to 29.7%) 697/2525	19.4% (17.6% to 21.3%) 426/2197	34.2% (32.3% to 36.1%) 1272/3720	20.5% (16.9% to 24.7%) 110/536	30.1% (27.6% to 32.7%) 544/1809	2.5% (1.1% to 4.9%) 8/322	23.1% (21.0% to 25.2%) 476/2063
1 or more neurological symptoms	67.5% (66.4% to 68.7%) 4231/6264	40.6% (38.0% to 43.4%) 897/2207	53.3% (50.6% to 56.1%) 1483/2783	39.1% (36.5% to 41.9%) 809/2067	58.4% (56.0% to 60.9%) 2284/3910	23.1% (19.0% to 27.8%) 112/485	55.4% (52.1% to 58.8%) 1045/1887	4.4% (2.4% to 7.4%) 14/318	40.0% (37.3% to 42.8%) 830/2075

Follow-up defined in months post hospital discharge. Data are reported as observed prevalence and 95% confidence intervals, assuming a Gamma distribution. (–): confidence interval not defined due to no cases reported. First assessment of symptoms was defined as the first time a patient completed a follow-up survey. Follow-up assessment of symptoms was defined as a patient who already completed their first survey and are being surveyed again. Estimates are stratified by first versus repeat symptom assessment to examine symptom persistence following hospital discharge. Observed prevalence at acute hospitalization is also presented

**Fig. 1:**
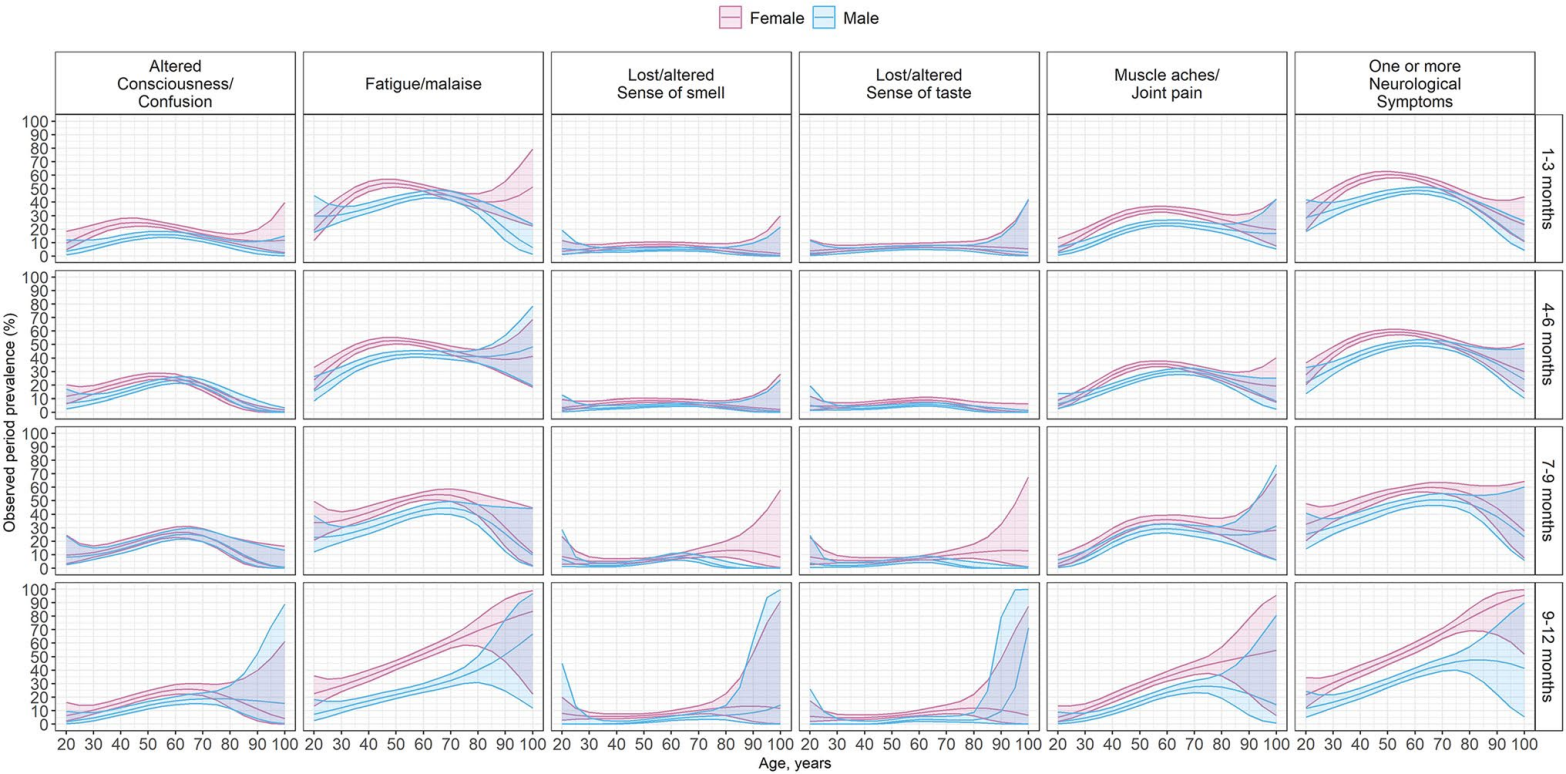
Observed prevalence by age and sex, months following hospital discharge. Observed prevalence of common neurological symptoms by age at hospitalization*, sex, and survey follow-up time. Follow-up time is defined in months since hospital discharge. Seizures excluded due to insufficient cases reported. Estimates presented are from a logistic regression model fitted to each symptom as the dependent variable, with fixed effects for sex (categorical), age (continuous) and their interaction. Age was modelled using polynomial terms up to order 3

The main findings of this international, multicenter, observational follow-up study are that (1) all symptoms declined over follow-up time, except confusion and insomnia; (2) among symptoms not evaluated during acute hospitalization, estimates of muscle weakness, headache, problems with balance, paresthesia, problems speaking/ communicating, dizziness, problems seeing, tremor, tinnitus, fainting, and problems swallowing/chewing gradually declined from 1–3 months to 10–12 months follow-up, whereas estimates of loss of sensation, erectile dysfunction, and problems sleeping gradually increased over time.

To the best of our knowledge, this is one of the largest cohorts (11,357 subjects) reporting post-COVID-19 neurological symptoms investigating.

It is noteworthy that the median age of our cohort was 56 years (IQR = 45 to 67), suggesting that most of them were actively working before acute COVID-19. This has been previously highlighted in another cohort, with many patients having difficulty returning to work and previous activities, with significant socio-economic consequences as documented previously how long-COVID-19 impacted employment and working full-time [6].

Long-term and cognitive symptoms have been previously reported in COVID-19 subjects [5]. Previous large investigations reported that around 10% of subjects diagnosed with acute COVID-19 still had symptoms after 1-year of follow-up [7]. In our cohort, fatigue/malaise, followed by muscle aches/joint pain were the most frequent neurological symptoms reported at the time of acute illness, with a trend toward decreasing frequency at each follow-up, suggesting gradual recovery of functional activities and progressive rehabilitation [8].

Interestingly, we noted a different recovery between symptoms of the central and peripheral nervous systems. Anosmia and ageusia (peripheral) disappeared completely, whereas confusion and insomnia (central) persisted. The occurrence of anosmia and ageusia is supposed to be caused by a local inflammatory response to SARS-CoV-2 infection targeting peripheral neurons. On the other hand, several systemic factors have been identified as possible responsible for central nervous system symptoms, some of them difficult to recover, including hypoxia, cerebrovascular illness, immune response, medical resources and treatments, social isolation, psychological repercussions of the pandemic, and the worry of spreading the sickness [9].

Many COVID-19 survivors were bed bound with persistent disconnection from their environment during their acute illness/hospital admission. Contributors of this status included prolonged use of sedatives and delirium during hospitalization. This may explain why we observed a trend toward increased estimates of erectile dysfunction, loss of sensation, and problems sleeping. Other common explanations for persistent neurological symptoms include residual tissue damage, viral persistence, and chronic inflammation [10], but also increasing age in patients with an underlying disease [11].

The major strength of this study is the description of the prognosis of the disease with inclusion of many subjects across 16 countries, highly representative of the general population, up to 12 months following hospital discharge [6]. Nevertheless, it is worth noting that only 22 patients were from the Americas, thus consideration of our findings in this population should be careful. Inconsistent data capture (sampling bias) and lack of rigorous definitions are a limitation. Indeed, a protocol for 12-months follow-up was not systematically implemented in all participating centers, and the data captured is rather driven by current clinical practice in each site. However, we use a large cohort with pragmatic data capturing across multiple countries that represents real-world reported observations. Lack of comparison group is a further limitation of this study. Moreover, a cluster analysis early on in our study was deemed infeasible based on the complex patterns of missing data observed (e.g., non-response to selected symptoms at initial hospitalization and/or survey follow-up; loss to follow-up). Finally, our results are based on the analysis of individual symptom prevalence. A more in-depth approach to analysis would be to instead consider patterns in co-occurring symptoms over time, for example, by cluster analysis. Given complexities in the data arising from differences in individual follow-up time and loss to follow-up, this option could not be explored. The application of clustering algorithms should be considered by future studies pending data availability.

Long-COVID-19 symptoms are common and persist over time. Registry activities and rehabilitation protocols should be implemented to define the burden of long-COVID-19 globally with standardized definitions and data capture instruments and ensure adequacy of resource distribution.

## Availability of data and material:

The data that underpin this analysis are highly detailed clinical data on individuals hospitalized with COVID-19. Due to the sensitive nature of these data and the associated privacy concerns, they are available via a governed data access mechanism following review of a data access committee. Data can be requested via the IDDO COVID-19 Data Sharing Platform (http://www.iddo.org/covid-19). The Data Access Application, Terms of Access and details of the Data Access Committee are available on the website. Briefly, the requirements for access are a request from a qualified researcher working with a legal entity who have a health and/or research remit; a scientifically valid reason for data access which adheres to appropriate ethical principles. The full terms are at https://www.iddo.org/document/covid-19-data-access-guidelines. A small subset of sites who contributed data to this analysis have not agreed to pooled data sharing as above. In the case of requiring access to these data, please contact the corresponding author in the first instance who will look to facilitate access.

### Supplementary Information

Below is the link to the electronic supplementary material.

Supplementary file1 (DOCX 1077 KB)

## References

[1] Levine RL (2022). Addressing the long-term effects of COVID-19. JAMA.

[2] World Heart Organization (WHO). Post COVID-19 condition (Long COVID) [Internet]. December 7. 2022. https://www.who.int/europe/news-room/fact-sheets/item/post-covid-19-condition. Accessed 13 Jun 2023

[3] Lopez-Leon S, Wegman-Ostrosky T, Perelman C, Sepulveda R, Rebolledo PA, Cuapio A (2021). More than 50 long-term effects of COVID-19: a systematic review and meta-analysis. Sci Rep.

[4] Chen C, Haupert SR, Zimmermann L, Shi X, Fritsche LG, Mukherjee B (2022). Global Prevalence of Post-Coronavirus Disease 2019 (COVID-19) Condition or Long COVID: a meta-analysis and systematic review. J Infect Dis.

[5] Premraj L, Kannapadi NV, Briggs J, Seal SM, Battaglini D, Fanning J (2022). Mid and long-term neurological and neuropsychiatric manifestations of post-COVID-19 syndrome: a meta-analysis. J Neurol Sci [Internet]..

[6] Suran M (2023). Long COVID linked with unemployment in new analysis. JAMA.

[7] Robineau O, Zins M, Touvier M, Wiernik E, Lemogne C, de Lamballerie X (2022). Long-lasting symptoms after an acute COVID-19 infection and factors associated with their resolution. JAMA Netw Open.

[8] Mohabbat AB, Mohabbat NML, Wight EC (2020). Fibromyalgia and chronic fatigue syndrome in the age of COVID-19. Mayo Clin Proc Innov Qual Outcomes.

[9] Sobrino-Relaño S, Balboa-Bandeira Y, Peña J, Ibarretxe-Bilbao N, Zubiaurre-Elorza L, Ojeda N (2023). Neuropsychological deficits in patients with persistent COVID-19 symptoms: a systematic review and meta-analysis. Sci Rep.

[10] Yong SJ (2021). Persistent Brainstem Dysfunction in Long-COVID: a hypothesis. ACS Chem Neurosci.

[11] Kennedy M, Helfand BKI, Gou RY, Gartaganis SL, Webb M, Moccia JM (2020). Delirium in older patients with COVID-19 presenting to the Emergency Department. JAMA Netw Open.

