# Supplementary material for: Long-term neurological symptoms after acute COVID-19 illness requiring hospitalization in adult patients: insights from the ISARIC-COVID-19 follow-up study
Source: J Neurol. 2023 Dec 6;271(1):79–86. doi: 10.1007/s00415-023-12133-y (PMC10769963; doi:10.1007/s00415-023-12133-y)
Supplement: Supplementary file 1 — Supplementary file1 (DOCX 1077 KB) [file 415_2023_12133_MOESM1_ESM.docx]

**SUPPLEMENTARY MATERIAL (SM)**

**Table of content:**

SM Item S1. COVID-19 long term protocol. [page 2]

SM Item S2. Study flowchart. [page 3]

SM Item S3**.** Distribution of initial acute hospitalizations among Long-COVID protocol respondents. [page 4]

SM Item S4. Baseline characteristics. [page 5]

SM Item S5. Period prevalence of neurological symptoms evaluated at acute hospitalization Observed prevalence by time period, month post-discharge (%) [page 6]

SM Item S6. Observed prevalence and persistence of neurological symptoms over survey follow-up post hospital discharge [page 7-13]

SM Item S7. Observed prevalence and persistence of neurological symptoms over survey follow-up post-acute illness [page 14]

**Supplementary Material Item S1. COVID-19 long term protocol.**

The following link refers to the COVID-19 long term protocol: https://isaric.org/research/covid-19-clinical-research-resources/covid-19-long-term-follow-up-study/#:~:text=Long%20term%20follow%2Dup%20protocol,a%20range%20of%20validated%20tools

The CRF of this survey include:

**Tier 1 Initial Freestanding follow up survey**– use for any person (hospitalized or not hospitalized) at the first follow up assessment

OR

**Tier 1 Initial Follow up survey**– use for a person with a Core or Rapid CRF completed during an acute hospital admission at the first assessment

Tier 1 Ongoing survey – for additional assessment time points

**Supplementary Material Item S2: Study flowchart:** Neurological signs and symptoms evaluated at acute hospitalization: Altered consciousness/confusion, Fatigue/Malaise, Lost/Altered sense of smell (Anosmia), Lost/Altered sense of taste (Ageusia), Muscle aches/joint pain, Seizures. For each survey follow-up period, the number of respondents is given along with the median and lower/upper quantiles for days since hospital discharge.


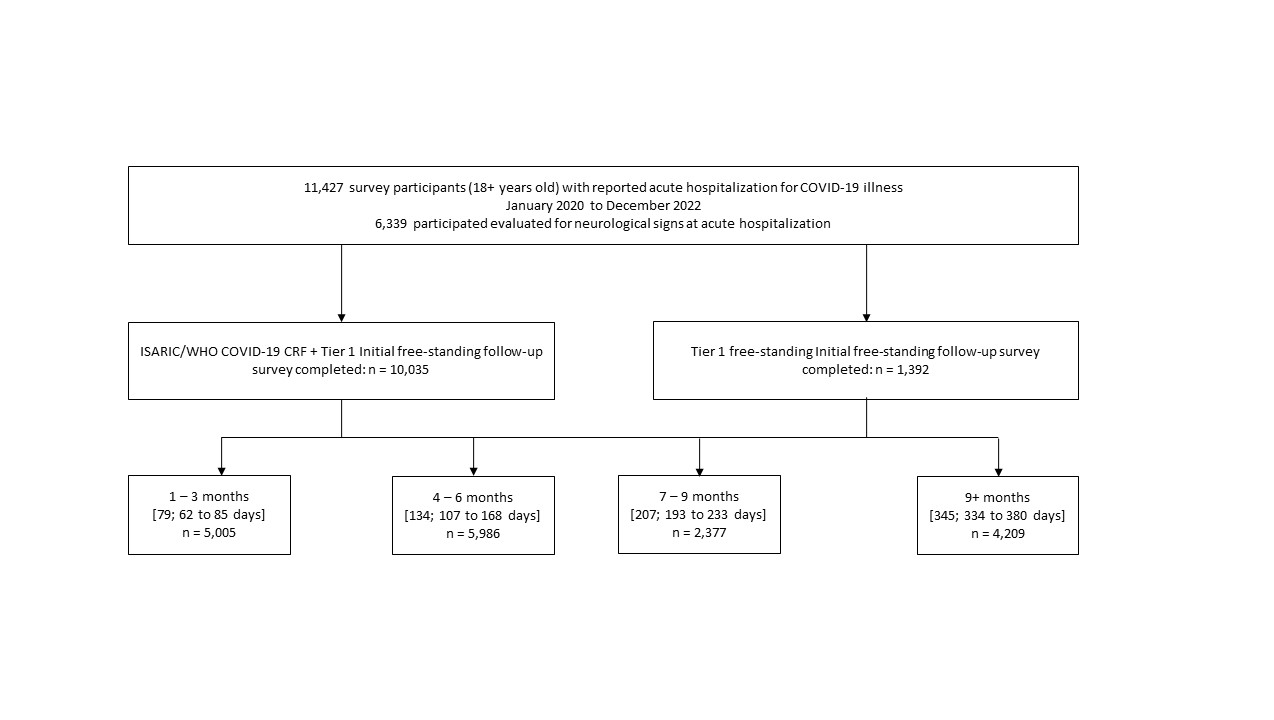


**Supplementary Material Item S3: Distribution of initial acute hospitalizations among Long-COVID protocol respondents.** Frequencies are stratified by the availability of neurological signs and symptoms evaluates at disease onset/hospital admission.


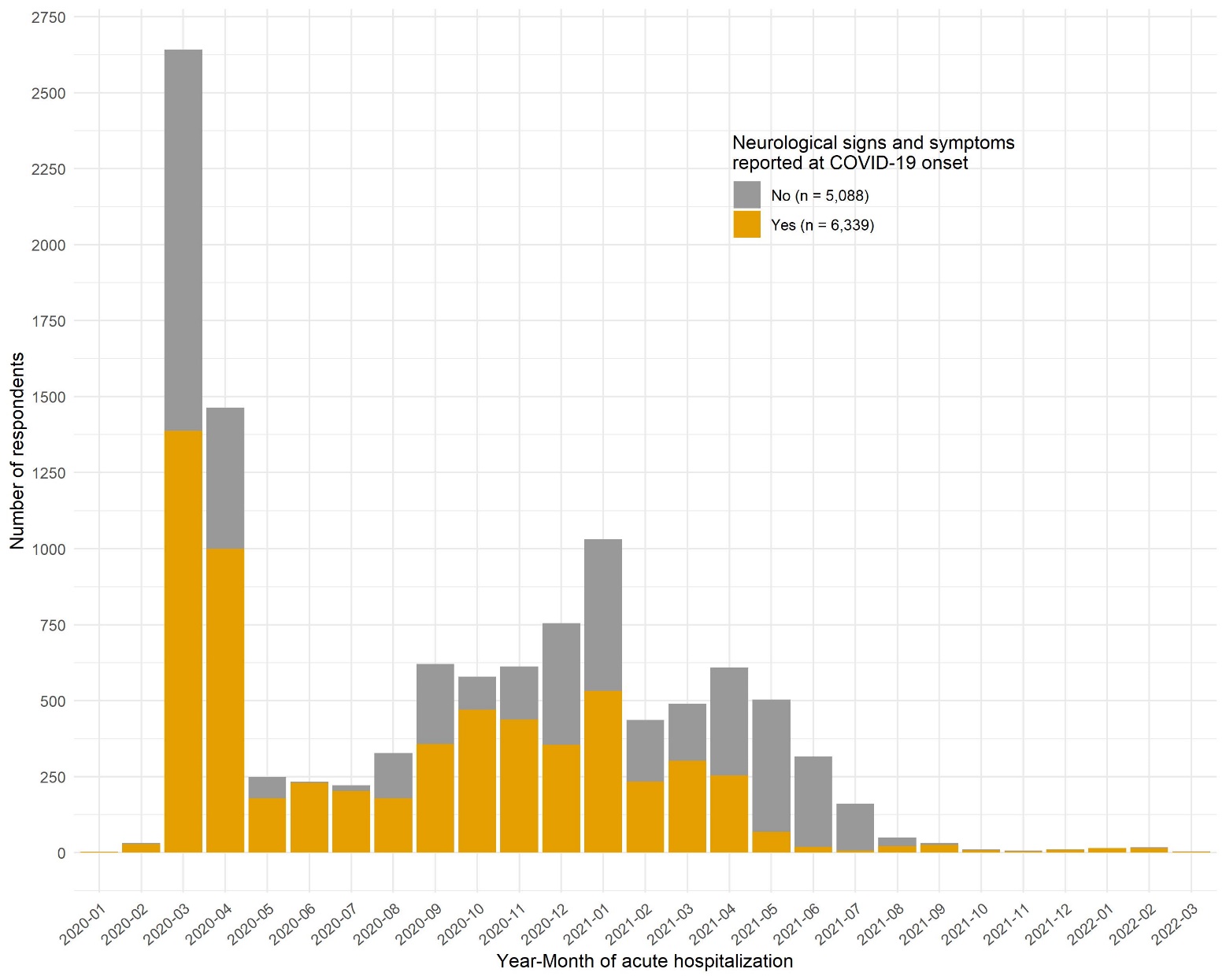


**Supplementary Material Item S4: Baseline characteristics.** Characteristics are summarised for the full study cohort. Continuous variables are summarised by the median and interquartile range (Q1 to Q3), and mean and standard deviation (SD). Categorical variables are summarised by the number of events/total respondents and percentage.

| **Characteristic** | **All survey respondents** |
| --- | --- |
| Age at hospitalization, years: Median (IQR) | 56 (45 to 67) |
| Age at hospitalization, years: Mean (SD) | 55.8 (15.3) |
| Female | 4,806 (41.9%) |
| Admitted to ICU during acute hospitalization | 3 024 (26.8%) |
| Hospital length of stay, days: Median (IQR) | 9 (6 to 14) |
| Hospital length of stay, days: Mean (SD) | 12.6 (12.3) |
| Geographic region |  |
| Africa | 2,273 (19.8%) |
| Americas | 23 (0.2%) |
| Asia | 1,860 (16.2%) |
| Europe | 7,320 (63.8%) |
| Country income classification |  |
| High Income | 7,437 (64.8%) |
| Upper-Middle | 2,251 (19.6%) |
| Low-Middle | 1,785 (15.6%) |
| Low | 3 (<0.1%) |

**Supplementary Material Item S5: Period prevalence of neurological symptoms evaluated at acute hospitalization.** Summary data are reported as the total number of observed cases divided by the total number of participants evaluated and as an unadjusted estimate with a 95% confidence interval (CI)

| **Symptom** | 1-3 months | 4-6 months | 7-9 months | 9+ months |
| --- | --- | --- | --- | --- |
| 1+ neurological symptoms | 47.7% (45.8% to 49.7%) 2,381/4,992 | 51.8% (49.9% to 53.6%) 3,095/5,980 | 48.8% (46.0% to 51.6%) 1,158/2,375 | 49.4% (47.3% to 51.6%) 2,079/4,209 |
| Altered consciousness/confusion | 14.3% (13.1% to 15.6%) 531/3,712 | 21.4% (20.1% to 22.7%) 1,004/4,695 | 21.7% (19.6% to 24.0%) 400/1,842 | 25.2% (23.7% to 26.8%) 1,049/4,156 |
| Fatigue/malaise | 45.4% (43.2% to 47.5%) 1,728/3,810 | 45.9% (44.1% to 47.9%) 2,270/4,943 | 41.2% (38.5% to 44.1%) 868/2,105 | 38.8% (36.9% to 40.7%) 1,609/4,152 |
| Lost/altered sense of smell | 6.2% (5.4% to 7.0%) 237/3,828 | 6.2% (5.5% to 6.9%) 306/4,944 | 6.6% (5.5% to 7.8%) 138/2,098 | 5.7% (5.0% to 6.5%) 237/4,165 |
| Lost/altered sense of taste | 6.8% (6.0% to 7.7%) 259/3,822 | 5.8% (5.2% to 6.5%) 287/4,943 | 5.8% (4.8% to 7.0%) 122/2,095 | 5.1% (4.5% to 5.9%) 214/4,172 |
| Muscle aches/joint pain | 23.2% (21.9% to 24.6%) 1,137/4,893 | 28.7% (27.4% to 30.1%) 1,699/5,920 | 27.9% (25.8% to 30.1%) 655/2,348 | 32.8% (31.1% to 34.5%) 1,376/4,200 |
| Seizures | 0.2% (0.0% to 0.4%) 4/2,591 | 0.4% (0.2% to 0.7%) 15/3,704 | 0.6% (0.3% to 1.2%) 10/1,592 | 0.5% (0.3% to 0.8%) 20/4,112 |

| **Supplementary Material Item S6 Observed prevalence and persistence of neurological symptoms over survey follow-up post hospital discharge**. (Not evaluated at acute hospitalization) | | | | |
| --- | --- | --- | --- | --- |
| **Symptom** | **Post-discharge follow-up,** | **Period prevalence of symptom** | **First assessment of symptom** | **Follow-up assessment of symptom** |
| Dizziness | 1-3 months | 7.5% (6.3% to 8.8%) 141/1,890 | 7.5% (6.3% to 8.8%) 141/1,890 | -- |
|  | 4-6 months | 8.2% (7.1% to 9.4%) 193/2,364 | 8.1% (6.8% to 9.7%) 126/1,550 | 8.2% (6.4% to 10.5%) 67/814 |
|  | 7-9 months | 4.8% (3.5% to 6.3%) 46/968 | 3.9% (2.4% to 6.0%) 20/513 | 5.7% (3.7% to 8.4%) 26/455 |
|  | 9+ months | 5.6% (4.7% to 6.6%) 138/2,455 | 3.7% (2.3% to 5.6%) 21/575 | 6.2% (5.1% to 7.5%) 117/1,880 |
| Erectile dysfunction | 1-3 months | 4.6% (3.6% to 5.8%) 75/1,627 | 4.6% (3.6% to 5.8%) 75/1,627 | -- |
|  | 4-6 months | 9.5% (8.2% to 10.9%) 200/2,112 | 10.8% (9.3% to 12.5%) 180/1,672 | 4.5% (2.8% to 7.0%) 20/440 |
|  | 7-9 months | 8.6% (6.8% to 10.6%) 84/982 | 9.9% (7.7% to 12.5%) 70/710 | 5.1% (2.8% to 8.6%) 14/272 |
|  | 9+ months | 11.0% (9.8% to 12.4%) 281/2,548 | 5.6% (4.0% to 7.5%) 44/791 | 13.5% (11.8% to 15.3%) 237/1,757 |
| Fainting/blackouts | 1-3 months | 1.7% (1.1% to 2.5%) 25/1,498 | 1.7% (1.1% to 2.5%) 25/1,498 | -- |
|  | 4-6 months | 1.7% (1.1% to 2.3%) 32/1,936 | 1.5% (1.0% to 2.2%) 26/1,741 | 3.1% (1.1% to 6.7%) 6/195 |
|  | 7-9 months | 1.6% (0.9% to 2.5%) 18/1,130 | 1.5% (0.8% to 2.5%) 14/958 | 2.3% (0.6% to 6.0%) 4/172 |
|  | 9+ months | 1.3% (0.9% to 1.9%) 30/2,312 | 1.2% (0.5% to 2.4%) 8/662 | 1.3% (0.8% to 2.0%) 22/1,650 |
| Headache | 1-3 months | 15.3% (14.2% to 16.4%) 747/4,892 | 15.3% (14.2% to 16.4%) 747/4,892 | -- |
|  | 4-6 months | 18.2% (17.1% to 19.3%) 1,078/5,936 | 19.3% (18.1% to 20.7%) 832/4,301 | 15.0% (13.2% to 17.0%) 246/1,635 |
|  | 7-9 months | 18.4% (16.7% to 20.2%) 431/2,346 | 21.8% (19.4% to 24.4%) 302/1,386 | 13.4% (11.2% to 16.0%) 129/960 |
|  | 9+ months | 18.8% (17.5% to 20.2%) 788/4,192 | 8.8% (6.8% to 11.3%) 64/724 | 20.9% (19.4% to 22.5%) 724/3,468 |
| Loss of sensation | 1-3 months | 0.6% (0.3% to 1.1%) 12/1,872 | 0.6% (0.3% to 1.1%) 12/1,872 | -- |
|  | 4-6 months | 0.7% (0.4% to 1.1%) 16/2,337 | 0.8% (0.4% to 1.4%) 12/1,521 | 0.5% (0.1% to 1.3%) 4/816 |
|  | 7-9 months | 1.0% (0.5% to 1.9%) 10/962 | 1.0% (0.3% to 2.3%) 5/509 | 1.1% (0.4% to 2.6%) 5/453 |
|  | 9+ months | 0.6% (0.3% to 1.0%) 15/2,454 | 0.8% (0.3% to 1.9%) 5/599 | 0.5% (0.3% to 1.0%) 10/1,855 |
| Muscle weakness | 1-3 months | 19.5% (17.8% to 21.2%) 510/2,618 | 19.5% (17.8% to 21.2%) 510/2,618 | -- |
|  | 4-6 months | 23.8% (22.2% to 25.4%) 880/3,705 | 26.6% (24.7% to 28.6%) 736/2,766 | 15.3% (12.9% to 18.1%) 144/939 |
|  | 7-9 months | 23.1% (20.8% to 25.6%) 360/1,558 | 26.7% (23.7% to 30.0%) 278/1,041 | 15.9% (12.6% to 19.7%) 82/517 |
|  | 9+ months | 21.5% (20.1% to 23.0%) 883/4,107 | 11.6% (9.2% to 14.5%) 80/689 | 23.5% (21.9% to 25.2%) 803/3,418 |
| Paraesthesia | 1-3 months | 9.0% (7.9% to 10.2%) 233/2,589 | 9.0% (7.9% to 10.2%) 233/2,589 | -- |
|  | 4-6 months | 16.0% (14.7% to 17.4%) 590/3,686 | 17.8% (16.3% to 19.5%) 492/2,758 | 10.6% (8.6% to 12.9%) 98/928 |
|  | 7-9 months | 15.8% (13.9% to 17.9%) 246/1,554 | 19.4% (16.9% to 22.3%) 202/1,039 | 8.5% (6.2% to 11.5%) 44/515 |
|  | 9+ months | 16.5% (15.2% to 17.8%) 675/4,100 | 8.8% (6.7% to 11.3%) 62/706 | 18.1% (16.7% to 19.5%) 613/3,394 |
| Problems seeing | 1-3 months | 7.3% (6.3% to 8.4%) 188/2,581 | 7.3% (6.3% to 8.4%) 188/2,581 | -- |
|  | 4-6 months | 10.0% (9.0% to 11.1%) 370/3,692 | 10.3% (9.1% to 11.5%) 284/2,770 | 9.3% (7.5% to 11.5%) 86/922 |
|  | 7-9 months | 10.9% (9.3% to 12.7%) 169/1,553 | 11.5% (9.5% to 13.8%) 120/1,043 | 9.6% (7.1% to 12.7%) 49/510 |
|  | 9+ months | 9.3% (8.4% to 10.3%) 381/4,095 | 5.0% (3.5% to 6.9%) 35/705 | 10.2% (9.2% to 11.3%) 346/3,390 |
| Problems sleeping | 1-3 months | 11.9% (10.5% to 13.4%) 259/2,184 | 11.9% (10.5% to 13.4%) 259/2,184 | -- |
|  | 4-6 months | 14.8% (13.4% to 16.3%) 395/2,668 | 17.7% (15.9% to 19.7%) 328/1,851 | 8.2% (6.4% to 10.4%) 67/817 |
|  | 7-9 months | 16.0% (13.8% to 18.4%) 187/1,172 | 20.5% (17.3% to 24.1%) 146/713 | 8.9% (6.4% to 12.1%) 41/459 |
|  | 9+ months | 23.4% (21.9% to 25.1%) 850/3,628 | 34.8% (31.8% to 38.1%) 475/1,364 | 16.6% (14.9% to 18.3%) 375/2,264 |
| Problems speaking or communicating | 1-3 months | 7.8% (6.7% to 8.9%) 203/2,612 | 7.8% (6.7% to 8.9%) 203/2,612 | -- |
|  | 4-6 months | 11.0% (9.8% to 12.3%) 319/2,893 | 10.9% (9.7% to 12.3%) 291/2,664 | 12.2% (8.1% to 17.7%) 28/229 |
|  | 7-9 months | 10.7% (9.1% to 12.6%) 148/1,381 | 10.9% (9.2% to 13.0%) 132/1,207 | 9.2% (5.3% to 14.9%) 16/174 |
|  | 9+ months | 13.9% (12.4% to 15.5%) 328/2,360 | 4.0% (2.7% to 5.8%) 28/692 | 18.0% (16.0% to 20.1%) 300/1,668 |
| Problems swallowing or chewing | 1-3 months | 1.3% (0.9% to 2.0%) 25/1,859 | 1.3% (0.9% to 2.0%) 25/1,859 | -- |
|  | 4-6 months | 0.9% (0.6% to 1.4%) 21/2,328 | 0.7% (0.4% to 1.3%) 11/1,515 | 1.2% (0.6% to 2.3%) 10/813 |
|  | 7-9 months | 0.6% (0.2% to 1.3%) 6/969 | 1.0% (0.3% to 2.3%) 5/514 | 0.2% (0.0% to 1.2%) 1/455 |
|  | 9+ months | 0.7% (0.4% to 1.1%) 16/2,452 | 0.8% (0.3% to 2.0%) 5/596 | 0.6% (0.3% to 1.1%) 11/1,856 |
| Problems with balance | 1-3 months | 9.7% (8.6% to 11.0%) 251/2,583 | 9.7% (8.6% to 11.0%) 251/2,583 | -- |
|  | 4-6 months | 14.8% (13.5% to 16.0%) 545/3,694 | 16.4% (14.9% to 18.0%) 454/2,764 | 9.8% (7.9% to 12.0%) 91/930 |
|  | 7-9 months | 14.5% (12.7% to 16.5%) 226/1,557 | 17.3% (14.9% to 20.0%) 181/1,045 | 8.8% (6.4% to 11.8%) 45/512 |
|  | 9+ months | 14.7% (13.6% to 16.0%) 603/4,093 | 8.7% (6.7% to 11.2%) 61/699 | 16.0% (14.7% to 17.4%) 542/3,394 |
| Tinnitus | 1-3 months | 2.2% (1.2% to 3.6%) 15/683 | 2.2% (1.2% to 3.6%) 15/683 | -- |
|  | 4-6 months | 3.6% (2.2% to 5.7%) 19/522 | 3.9% (2.3% to 6.1%) 18/466 | 1.8% (0.0% to 9.9%) 1/56 |
|  | 7-9 months | 1.8% (0.8% to 3.5%) 9/489 | 1.0% (0.3% to 2.5%) 4/413 | 6.6% (2.1% to 15.4%) 5/76 |
|  | 9+ months | 2.0% (1.1% to 3.5%) 13/635 | 1.5% (0.6% to 2.9%) 8/536 | 5.1% (1.6% to 11.8%) 5/99 |
| Tremor | 1-3 months | 5.6% (4.5% to 6.9%) 83/1,485 | 5.6% (4.5% to 6.9%) 83/1,485 | -- |
|  | 4-6 months | 10.3% (8.9% to 11.9%) 198/1,917 | 10.4% (8.9% to 12.0%) 179/1,724 | 9.8% (5.9% to 15.4%) 19/193 |
|  | 7-9 months | 8.4% (6.8% to 10.2%) 95/1,137 | 8.2% (6.5% to 10.2%) 79/965 | 9.3% (5.3% to 15.1%) 16/172 |
|  | 9+ months | 10.0% (8.8% to 11.4%) 234/2,339 | 4.4% (3.0% to 6.3%) 31/704 | 12.4% (10.8% to 14.2%) 203/1,635 |

**Supplementary Material Item S7: Observed prevalence and persistence of neurological symptoms over survey follow-up post-acute illness.**

**
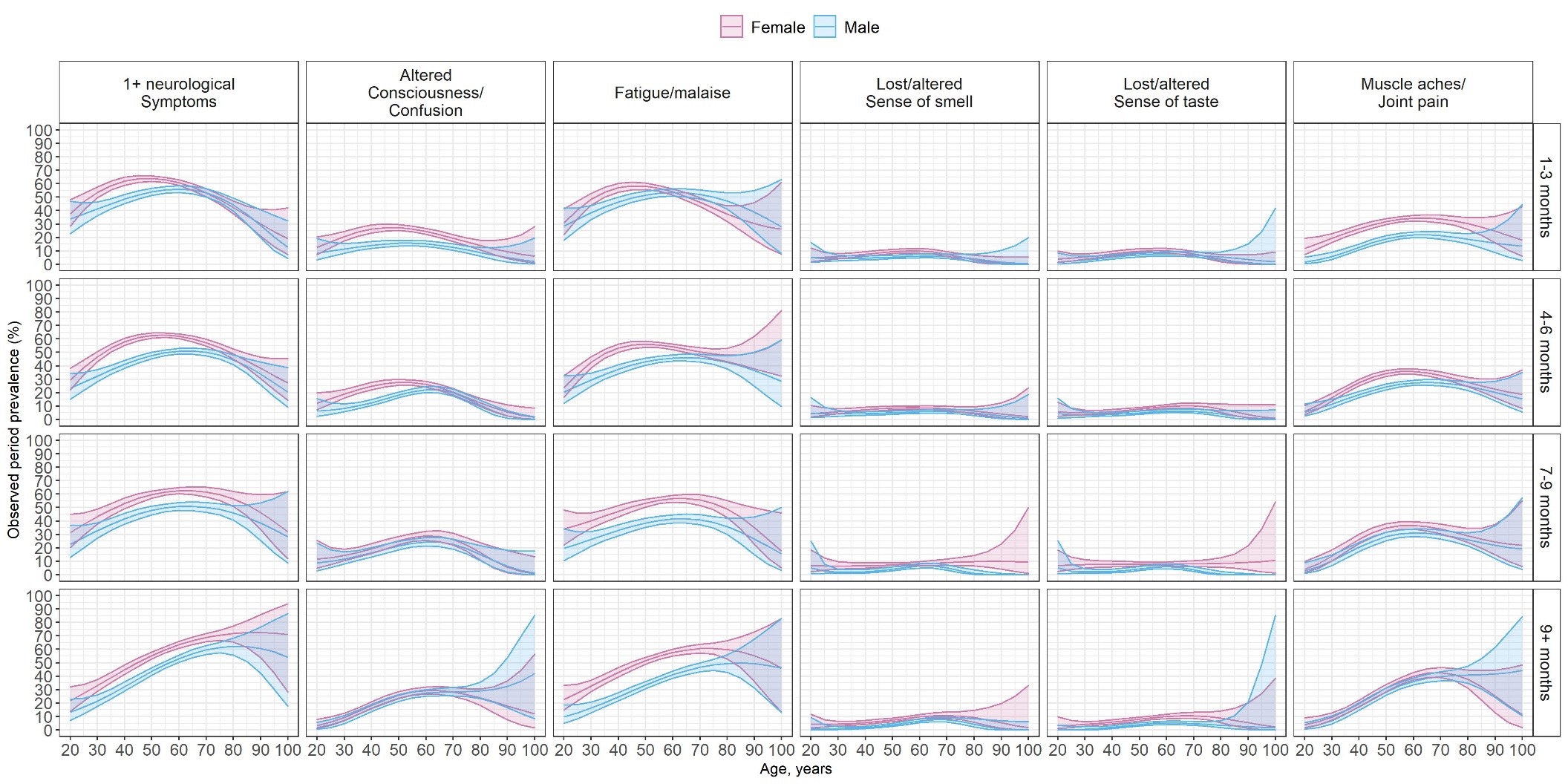
**

Seizures excluded due to insufficient cases reported
